# Impact of dental pulp cells-derived small extracellular vesicles on the properties and behavior of dental pulp cells: an in-vitro study

**DOI:** 10.1186/s12903-025-06031-0

**Published:** 2025-05-10

**Authors:** Dina A. Hammouda, Alaa M. Mansour, Ahmed R. Zaher, Mohammed E. Grawish

**Affiliations:** 1 Department of Oral Biology, Faculty of Dentistry, Mansoura University, Mansoura, 35511 Egypt; 2 Department of Oral Biology, Faculty of Oral and Dental Medicine, Delta University for Science and Technology, Dakahlia, Egypt

**Keywords:** sEVs, DPCs, MTA, Dental pulp, Regenerative dentistry, Pulp capping

## Abstract

**Background:**

Dental pulp cells-derived small extracellular vesicles (DPCs-sEVs) had shown immunomodulatory, anti-inflammatory, and tissue function restorative abilities. Therefore, DPCs-sEVs should be considered as a promising regenerative tool for dentin-pulp complex or whole pulp regeneration. This study aimed to evaluate the effect of DPCs-sEVs on the proliferation rate, migration capability, and expression pattern of DPCs for osteo/odontogenic gene markers in comparison with mineral trioxide aggregate (MTA).

**Methods:**

DPCs-sEVs were isolated from rats’ incisors by ultracentrifugation technique. Immunophenotypic characterization, morphology, size, and protein concentration of DPCs-sEVs were monitored and analyzed using flow cytometry (FC), transmission electron microscopy (TEM), nanoparticle tracking analysis (NTA), and bicinchoninic acid assay (BCA). In addition, the TSG101, CD63, and the cytosolic protein syntenin of sEVs markers were immunodetected using Western blotting. Cell cultures of DPCs from the third passage were left untreated and considered as a control (group I), whereas other cultured cells were treated with 50 µg/mL DPCs-sEVs (group II), 0.2 mg/mL MTA extract (group III), or their combination (50 µg/mL DPCs-sEVs + 0.2 mg/mL MTA extract (group IV). 3-(4, 5-dimethylthiazol-2-yl)-2, 5-diphenyl tetrazolium bromide (MTT) assay, transwell migration assay, and real-time polymerase chain reaction were used for assessing proliferation, migration, and specific gene expression patterns.

**Results:**

The DPCs-sEVs increased DPCs proliferation, and MTA enhanced their effects. The viability and proliferative capacity of DPCs treated with 50 µg/mL DPCs-sEVs + 0.2 mg/mL MTA-conditioned medium was significantly higher when compared with the other groups. The cell migration was more prominent in the group treated with 0.2 mg/mL MTA-conditioned medium than in the group treated with 50 µg/mL DPCs-sEVs. DPCs treated with 50 µg/mL DPCs-sEVs + 0.2 mg/mL MTA extract showed a significant increase in the migration ability of DPCs in comparison with other ones. Moreover, the combination group showed the greatest expression of dentin sialophosphoprotein (D*spp)*, osteocalcin (O*cn*), collagen type I (C*ol1*), and runt-related transcription factor 2 (R*unx2*).

**Conclusion:**

MTA and sEVs together could be a powerful combination for regenerative endodontics.

**Clinical trial number:**

Not applicable.

**Supplementary Information:**

The online version contains supplementary material available at 10.1186/s12903-025-06031-0.

## Introduction

The dental pulp is a soft vascular tissue present within the tooth, and it is encircled by mineralized tissues, which are enamel, dentin, and cementum. Both pulp and dentin develop from the same origin [1]. Although the dentin-pulp complex has a continuous ability to form dentin, either secondary or tertiary dentin, its regenerative ability is considered limited [2]. Preservation of the pulp vitality is the main goal in dental treatment, as it is a critical factor in long-term tooth survival [3]. Direct pulp capping is a vital pulp therapy aimed at maintaining pulp vitality by placing a biocompatible material directly over the exposed pulp to seal the pulp and prevent pulpal infection, allowing the pulp tissue repair by stimulating the DPCs to form reparative dentin [4, 5]. For many decades, calcium hydroxide [Ca(OH)_2_] was considered the material of choice among the various available pulp-capping agents, and it has been widely used in clinical practice for direct pulp capping. Despite it being regarded as the gold standard for pulp capping, considerable limitations were reported, such as degradation over time, poor sealing ability, high solubility in oral fluids, pulp chamber obliteration, and the presence of tunnels in dentin bridges [6].

Abou ElReash et al. [7] concluded that iRoot-BP-Plus, MTA-HP and ACTIVA promoted human dental pulp stem cells (DPSCs) attachment, proliferation and mineralization in the laboratory in vitro setting [7]. In addition, Zaen El-Din et al. [8] found that tricalcium silicate-based cements [mineral trioxide aggregate (MTA) and Biodentine] and nano-hydroxyapatite are potential alternatives to Ca(OH)_2_ in vital pulp therapy following accidental pulp exposure in the laboratory and in vivo setting [8]. The results of a systematic review and meta-analysis demonstrated that the clinical outcome of direct pulp capping with MTA is superior to Ca(OH)_2_ [9]. MTA has been shown to exhibit faster bridging qualities than Ca(OH)_₂_ despite its long setting time, difficulty in handling, and high cost [10]. This might explain its in-situ success as an endodontic biomaterial. Therefore, an alternative gold standard MTA, has been introduced to overcome the drawbacks of Ca(OH)_2_.

The concept of ‘tissue engineering’ has rapid progress and depends on combining stem cells, biomaterials, and growth factors [11]. One of dental pulp cells (DPCs) is DPSCs. They are mesenchymal stem cells that are derived from neural crests. These DPSCs were shown to have the ability to generate ectopic dentin and related pulp tissue in vivo as well as to differentiate into neural-like cells in vitro. These recently discovered cells are a clonogenic and fast-proliferating population of mesenchymal stem cells (MSCs), according to follow-up research. MSCs differentiate into chondrocytes, adipocytes, and osteoblasts [12]. It has received a lot of attention because of their therapeutic value, which has been demonstrated for both oral and systemic disorders. Additionally, the immunomodulatory properties of DPSCs make them an important cell source for cell-based therapy of immune and inflammation-related diseases [13].

Recently, small extracellular vesicles (sEVs) have been suggested as a promising cell-free therapeutic alternative for stem cells [14]. sEVs are biological nanoscale spherical lipid bilayer vesicles. They contribute to cell-cell communication, antigen presentation, immune response modulation, and epigenetic reprogramming of recipient cells [15]. sEVs have the same therapeutic benefits as their parent cells; in addition, they are devoid of the parent cells’ shortcomings, providing a safer alternative approach over cell-based therapy with storage convenience [16]. The application of sEVs in different medical fields has gained a high interest as an excellent promising cell-free therapy. Dental pulp stem cells-derived small extracellular vesicles (DPSCs-sEVs) are highly attractive in regenerative medicine as they exhibit marked osteogenic/odontogenic induction ability and easy availability [17]. Compared to MSCs, sEVs possess unique advantages such as nanoscale size, low side effects, easy obtainability, excellent biocompatibility, low immunogenicity, high specificity, and high drug loading capacity [18]. sEVs derived from human exfoliated deciduous teeth and bone marrow mesenchymal stem cells (BMSCs) have been shown to promote periodontal tissue regeneration and repair of alveolar bone defects in rats [19, 20]. According to that, this research was performed to assess the effect of using DPCs-sEVs, MTA, and the combination (DPCs-sEVs/MTA) on DPCs proliferation, migration, and specific genes expression. In addition, we attempted to investigate the null hypothesis of no difference between the effectiveness of applying DPCs-sEVs, MTA, and DPCs-sEVs/MTA on DPCs against the alternative one of a difference.

## Materials and methods

### Animals

**Fig. 1:**
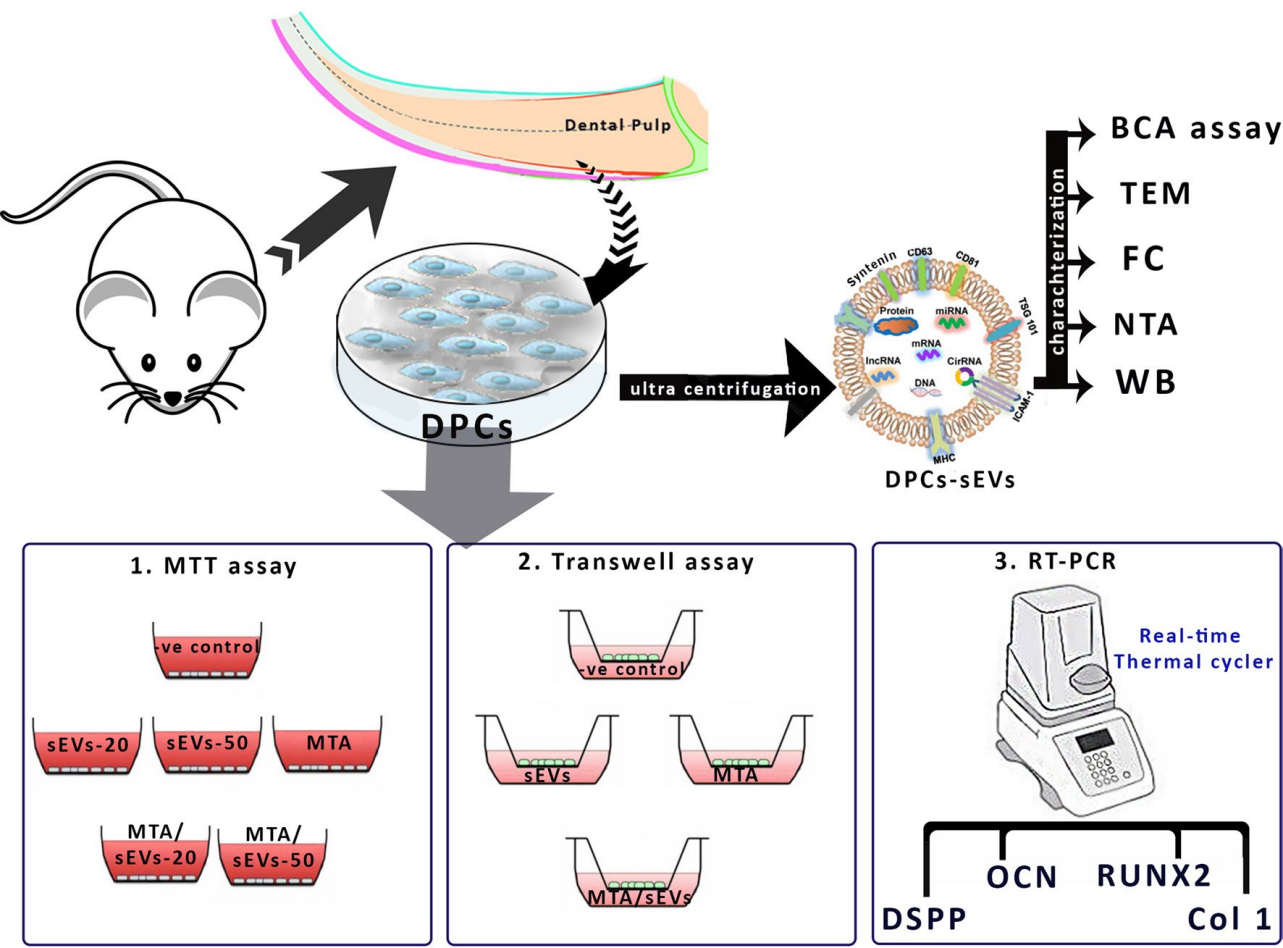
schematic diagram showing the study design. The pulp tissues were harvested from incisors’ rats followed by isolating DPCs that were ultracentrifuged to isolate DPCs-sEVs. The sEVs were characterized using transmission electron microscope (TEM), western blotting (WB), nano tracking assay (NTA) and bicinchoninic acid (BCA) assay. To evaluate the effect of DPCs-sEVs on DPCs, in comparison to MTA; MTT assay, transwell migration assay and RT-PCR were performed

The ARRIVE Checklist (https://www.nc3rs.org.uk/arrive-guidelines) and the guidelines of the Animal Research: Reporting In Vivo Experiments were followed in performing this study. Regarding the use and care of animals for this experiment, the protocol was approved by the Mansoura University Animal Care and Use Committee with a code number (DENT.MS.23.05.4). Six-week-old male Wistar rats were purchased from the Medical Research Centre, Faculty of Medicine, Mansoura University (n = 5), housed in standard rat’ cages with free access to water and dry pellets. The study design and methodology was illustrated in Fig. 1.

### Isolation of DPCs

Using a precision vaporizer with an induction chamber and waste gas scavenger, 5% isoflurane in oxygen as an inhalational anesthetic was administered slowly and continued for 100 s until respiratory arrest occurred. Then, the chamber was flushed with oxygen only, and the rats were removed and decapitated using guillotine with a sharp blade to ensure euthanasia. Then, the oral cavities were sterilized with a povidone-iodine solution. The incisors were gently extracted using a scalpel and forceps, and they were washed with phosphate-buffered saline (PBS) with 2% penicillin/streptomycin, and they were split open to carefully detach the pulp tissues. Then, with the use of a scalpel, the pulp tissues were pooled together and had been minced out of 1–2 mm³ fragments.

The direct cell outgrowth method was used for the isolation of DPCs [21]. Briefly, the combined minced fragments were transferred into a 25 cm² cell culture flask (Greiner) containing Dulbecco’s modified eagle medium (DMEM), supplemented with 10% foetal bovine serum (FBS), 100 µg/mL streptomycin, 100 µg/mL penicillin, and 2 mM L-glutamine (Gibco, SERANA, UK), incubated at 37ºC with a 5% CO_₂_ environment. The complete culture media was changed every three days, and examination was carried out daily using an inverted light microscope (Labomed, USA). When the cell confluence reached 80%, subculturing was performed by removing the culture media and washing using Dulbecco’s PBS (SERANA, UK). Then, adherent cells were detached by adding a trypsin/EDTA solution containing 0.25% trypsin and 1 mM EDTA. An adequate volume of fresh serum-containing medium was added to inactivate the trypsin, and then centrifugation was performed to obtain the cell pellet. A cell count of 0.1 mm³ was performed using a hemocytometer.

The hemocytometer value was obtained by multiplying the average number of cells with the dilution factor by 10^3^. Cell subculture was performed at a plating density of 1 × 10^6^ cells using a newly labelled flask containing a measured volume of the previously warmed complete culture medium. After the third passage, cells were collected for immunophenotypic characterization.

### Characterization of DPCs

Flow cytometry (FC) served as the method for the comprehensive analysis and confirmation of DPCs phenotypic characteristics. Antibodies targeting specific human antigens, including CD90, CD73, and CD105 as positive markers, and CD34, CD14, and CD45 (BD Biosciences, USA) as negative markers, were employed for immunophenotyping. DPCs were trypsinized with Trypsin-EDTA, washed with PBS (Cat. number: 10010023 Gibco, Thermo Scientific, USA), and subsequently resuspended in FC staining buffer. Following this, 100 µl of cells (1 × 10^6^) were incubated with fluorescein isothiocyanate (FITC)-conjugated CD90, Phycoerythrin (PE)-conjugated CD73, PE-conjugated CD105, PE-conjugated CD34, FITC-conjugated CD14, or FITC-conjugated CD45 (BD Biosciences, USA) in the dark for 30 min at 4ºC. The stained cells were centrifuged, washed, and resuspended in stain buffer for measurement. Unstained stem cells served as a negative control. The expression profiles were evaluated using a CytoFLEX S Flow Cytometer (Beckman Coulter, USA), and the FC data were analyzed utilizing the CytExpert software.

### Isolation of DPCs-sEVs

The sEVs were obtained from the supernatants of the DPCs by ultracentrifugation of 1 × 10^6^ DPCs/ml and seeded in a 25 cm^2^ cell culture flask (Greiner). At 80% cell confluence, the complete culture medium was replaced with the one without FBS and with a 1% antibiotic mix for 48 h. The supernatant was centrifuged at 500 xg for 10 min at 4ºC to eliminate the floating cells, then a second centrifugation was performed at 2,000 xg for 10 min, followed by a third one done at 10,000 xg for 1 h at 4ºC. To eliminate the apoptotic bodies and cell debris, the supernatants were filtrated through 0.22 μm filters. Finally, the cell-free supernatants were ultracentrifuged (Beckman Coulter, USA) at 100,000 xg at 4ºC for 2 h and washed in serum-free medium, and they were subjected again to ultracentrifugation at 100,000 xg at 4ºC for 70 min to eliminate protein contamination [22].

### Nanoparticle tracking analysis using ZetaView software

The size, concentration, and zeta potential measurements were investigated with a ZetaView S/N 21–619, Software ZetaView (version 8.05.14 SP7), equipped with a laser wavelength of 520 nm at a temperature of 37ºC, pH 7.0, and viscosity 0.899237. Nanoparticles were illuminated by the laser, and their movement under Brownian motion was captured for 60 s. Then, three more repetitions of this technique were performed. The sEVs concentrations and size distribution were determined using NTA on each of the three recorded videos using NanoSight particle tracking software.

### The sEVs purity and protein quantification

To quantify the sEVs’ concentration, the BCA protein assay kit (Novagen) was utilized following the manufacturer’s instructions. The ratio of reagents used was 2:100, 4% cupric sulphate: BCA solution. 2 µl of double distilled water (ddH_₂O_) was used for the blank measurement, and 2 µl of each standard was measured in replicate to plot the standard curve (regression curve). A 2 µl sample volume of sEVs was used to measure the protein concentration of unknown samples. Concentration was calculated from the standard curve, and results were presented in mg/Dl [23]. To calculate sEVs purity, total particle concentration was calculated by multiplying particle concentration measured by NTA with the dilution factor and total volume of the medium used for the sEVs preparation. Total protein concentration was calculated by multiplying protein concentration measured by BCA with the total volume of the medium used for the sEVs preparation. The purity was calculated by dividing the total particle concentration by the total protein concentration [24]. A higher sEVs purity indicates a higher level of particles with negligible protein content or other non-sEVs particles.

### Characterization of DPCs-sEVs using TEM, FC and Western blotting (WB)

For sEVs’ morphological characterization, a life science transmission electron microscope (TEM, JOEL JEM-1220, Tokyo, Japan) was used. 10 µL of a 1 in 10 dilution of the samples was applied into Formvar/carbon-coated nickel TEM grids, and they were left incubating for 30 min. Then, deionized water was used to wash the grids twice. After that, the grids were dried and stained with phosphotungstic acid as per standard procedures [25].

Through FC, the isolated sEVs were characterized using a panel of three biomarkers, including “anti-CD34, anti-CD9, and anti-HSP70” monoclonal antibodies (ThermoFisher Scientific, USA). Briefly, 1 × 10^10^/ml of sEVs that were suspended in 100 µL of PBS, containing 2% exosome-depleted FBS, supplemented with phosphatase-inhibitor and protease-inhibitor, were stained with a panel of three primary antibodies. The antibodies were used with a dilution of 1:100. For staining of sEVs, 15 µL of sEVs suspension was added to 5 µL of diluted antibody (1:100) and incubated for 45 min in the dark, at room temperature. Furthermore, washing buffer (0.2 μm-filtered PBS + 2% exosome-depleted-FBS) was used for washing, and the sEVs were incubated with (1:100) of secondary goat anti-rabbit IgG (H + L) Alexa Fluor Plus for 15 min at room temperature (Thermo Fisher, USA). Finally, the sample was examined by flow cytometry.

The laser was set to run with a power adjustment that kept the fluorophore intensity well inside the detection range or run at maximum power (405 nm: 175 mW; 488 nm: 145 mW; 561 nm: 90 mW; 642 nm: 145 mW). FITC was measured in channel 2 (480–560 nm filter) for the CD9 and HSP70, and PE was detected in channel 3 (560–595 nm filter) for the CD34. All readings were acquired at 60x magnification collected at low flow rate. Data analysis was performed using Navios EX software.

The protein content of the sEVs’ samples was detected using WB. The western blot assay was performed firstly for CD63, CD81, and Syntenin in the Proteomics and Metabolomics unit, Children’s Cancer Hospital 57,357, Cairo, Egypt. Briefly, protein was isolated using protease cocktail inhibitors and Radioimmunoprecipitation assay buffer (1x RIPA). The total protein content was determined using the Bradford assay. 15 µg of protein was used as a sample. A Laemmli buffer (BioRad, USA) was mixed with the calculated protein and heated at 70 °C for 10 min. The samples were then loaded onto an SDS-PAGE gel and subjected to electrophoresis at a constant 130 V for 140 min. The composition of the stacking and resolving gels was a mixture of Millipore water, Tris (Sigma), 10% SDS (Roth), 10% Ammonium Persulfate, APS (30% Acrylamide, BioRad), and TEMED (Invitrogen). Protein molecular weights were estimated using the Page Ruler™ Prestained Protein Ladder from Thermo Fisher Scientific. After electrophoresis, a PVDF membrane (0.45 μm, Millipore) was prepared with a brief methanol treatment for 2–3 min. This membrane was then positioned in a Criterion Blotter from BioRad along with a filter pad and blot filter paper, and protein transfer was carried out at 100 V for 50 min. The membrane was subsequently blocked using a 5% milk solution (Sigma-Aldrich) for an hour. Targeting specific proteins, primary antibodies including CD81 (BioLegend), CD63 (BioLegend), and Syntenin (Abcam) were applied. These antibodies were diluted in 5% milk to 1:1,000. The membrane was incubated with these antibodies overnight at 4ºC with agitation. For detection, secondary antibodies-horseradish peroxidase-conjugated goat anti-rabbit IgG and goat F(ab’)2 anti-mouse IgG (both from Thermo Scientific) were used at dilutions of 1:5,000 and 1:10,000, respectively, and incubated for 1 h at room temperature. Visualization was achieved using the SuperSignal West FemtoMaximum sensitivity substrate (Thermo Fisher Scientific) and imaged and analyzed by a ChemiDoc MP System (Bio-Rad Laboratories) and Image Lab™ Software (Bio-Rad Laboratories). For more confirmation, western blotting was performed for the same sEVs samples in Bernd Giebel Research Lab, Institute of Transfusion Medicine, University Hospital Essen, Germany. TSG 101 (Sigma), CD63 (BioLegend), and Syntenin (Abcam) were detected following the protocol performed by Ludwig et al. [26]. The visualization for the membrane was done using a Chemostar Plus imager and software (Isogen Lifescience B.V.).

### Preparation of MTA-extract

The selected concentration used in our study and the method of preparing the Bio MTA (CERKAMED, Poland) extract was dependent on a previous study performed by Babaki et al. [27]. Briefly, in a laminar flow hood, 4 vials of Bio-MTA powder (each one was 0.14 g) and one liquid vial were used. The whole content of the MTA powder was put on a mixing plate and mixed with 8 drops of the BIO MTA liquid. Drops were added one by one while mixing till reaching the consistency of soft plasticine (modelling putty). Then, the compound was placed in a culture tube. The mixture was then allowed to set at room temperature for 24 h. After the complete setting, the mixture was ground to a fine powder. Then, 200 mg of the powder had been filtered using a 45 μm strainer and mixed with 1 mL of DMEM-LG. The suspension was incubated at 37ºC in a 95% humidified air of 5% CO_2_ atmosphere to allow the release of bioactive ingredients into the medium. After 7 days, the supernatant was filtered through a 2.5 μm syringe filter to form an “MTA extract” at the concentration of 200 mg/mL. The concentration of 0.2 mg/mL MTA extract was then prepared by the addition of complete culture media [27].

### Cell viability assay

Cell viability was evaluated by 3-(4, 5-dimethylthiazol-2-yl)-2, 5-diphenyl tetrazolium bromide (**MTT**)-based assay. Firstly, this test was conducted to determine the optimal sEVs concentration that may be utilized alone or in combination with MTA. The tested sEVs concentrations were 20 µg/mL and 50 µg/mL, according to previous studies [16, 28, 29]. The assay was then carried out to investigate how MTA, sEVs, and their combination affected the spread of DPCs. For preparing the MTT solution, MTT was mixed with 10 mg/mL water, 20 mg/mL ethanol, 5 mg/mL buffered salt solutions, and culture media using a vortex, after that, the solution was filtered. DPCs were incubated in 96-well plates at a concentration of 4 × 10^3^ cells per well in a 200 µL culture medium. After that, the cells were grouped as follows: Group I: represents the control cells (untreated), Group II: DPCs treated with 0.2 mg/mL MTA extract, Group III: DPCs plates treated with 20 and 50 µg/mL DPCs-sEVs to compare the two concentrations and, Group IV: DPCs treated with 20 and 50 µg/mL DPCs-sEVs combined with 0.2 mg/mL MTA-extract.

After the 7- and 14-days incubation periods, 20 L of MTT solution (5 mg/mL) was used to replace the culture medium. Then, the wells were maintained at 37ºC for 4 h with 5% CO_₂_. After 4 h, the MTT solution was removed. After that, 150 µL of dimethyl sulfoxide was added to each well to dissolve the formazan crystals. Finally, a microplate reader at 545 nm was used to determine the optical density values [27].

### Cell migration assay

The transwell migration assay was used to evaluate the DPCs migratory behavior after being co-cultured with MTA and DPCs-sEVs either individually or in combination. The test was performed using the Boyden Chamber (Boyden of 8 mm calibre, ThermoFisher Scientific, Germany). In the biosafety cabinet, a 0.25% trypsin-EDTA solution (Gibco, USA) was used to detach the cultured cells from the culture flask, and then an adequate volume of media with serum was added to stop the trypsin activity. Then, centrifugation at 200 xg for 10 min was performed, and the existing media was aspirated, leaving the cell pellet, which was further resuspended in 1 mL of serum-free cell culture media. To the upper compartment, 100 µL of cell suspension was plated on top of the filter membrane. The cells were incubated for 10 min at 37ºC and 5% CO_₂_ to allow cells to settle down. To the lower compartment, 600 µL culture medium, 600 µL medium containing 0.2 mg/mL MTA, 600 µL medium containing 50 µg/mL DPCs-sEVs, or 600 µL medium containing both 50 µg/mL DPCs-sEVs and 0.2 mg/mL MTA were added, and they were considered as groups I, II, III, and IV, respectively.

After the adherence of cells in the upper compartment, the upper chambers were placed over the lower ones, and the chemoattractant liquid was placed in the bottom well to be in contact with the membrane in the upper well, forming a chemotactic gradient. DPCs were incubated in the transwell plate at 37ºC and 5% CO_2_ for 48 h, allowing them to migrate toward the underside of the insert filter. At the end of the incubation time, the transwell insert was taken out of the plate. A cotton-tipped applicator was used to gently eliminate the medium and the non-migrated cells. 600 µL of 70% ethanol was added into a well of a 24-well plate. The transwell insert was placed into 70% ethanol for 10 min to allow cell fixation. Then, the transwell insert was taken out of the plate, and the remaining ethanol at the top of the membrane was eliminated by a cotton-tipped applicator. The transwell insert was allowed to dry for 15 min. For staining, 600 µL of 0.2% crystal violet was added into the well, and the membrane was positioned into it and incubated at room temperature for 5–10 min.

The crystal violet at the top of the membrane was gently removed with a cotton-tipped applicator. Very carefully, the inset was dipped several times into distilled water to eliminate the excess crystal violet, and then it was left to dry. Subsequently, the membrane has been examined under an inverted microscope. The number of cells was counted in different fields of view. The average sum of cells that had migrated through the membrane toward the chemo-attractant and attached to the underside of the transwell membrane has been determined [30].

### Real-time polymerase chain reaction analysis

Real-time PCR was performed to evaluate mRNAs of O*cn* or B*glap*, R*unx2*, D*spp*, and C*ol1*. O*cn* and R*unx2* polynucleotide primers were designed and synthesized by Vivantis Technologies (ID: 181208361) with primer sequences described in Table 1. The B *actin* (Hs_ACTB), ID: QT000954231 primer was used as a housekeeping gene. One day before experimenting, an average of 1 × 10^7^ DPCs were seeded in a 6-well plate. Culture plates were incubated at 37ºC in an atmosphere of 5% CO_₂_ for 24 h. On the next day, cells were treated as previously described in the cell migration assay. Cells were maintained at 37ºC in an atmosphere of 5% CO_₂_ for 7 days as a differentiation period. The cells were examined daily. Every three days, the complete culture medium was changed for cell feeding and to eliminate the non-adherent cells. Cells were inspected regularly under an inverted microscope (Labomed, USA). After 7 days of incubation, cells were detached by using 0.25% trypsin, harvested, and washed two times with PBS, and the cell pellet was suspended in 1 mL of culture media to be used for gene expression analysis.

**Table 1:** Sequences of the forward and reverse primers of O*cn*, R*unx2*, D*spp*, C*ol 1* and B *actin*

Gene	Accession number	Forward primer	Reverse primer
O*cn*	NM_013414.1	GAG GGC AGT AAG GTG GTG AA	GTC CGC TAG CTC GTC ACA AT
R*unx2*	NM_001278483.2	CCA CCA CTC ACT ACC ACA CG	GGA CGC TGA CGA AGT ACC AT
D*spp*	NM_012790	TTCTCCTACTCAGCCCATTTTA	CCATCGTGACCGTATGTTTCTA
C*ol1*	NM_053356.2	TGTTCGTGGTTCTCAGGGTAG	TTGTCGTAGCAGGGTTCTTTC
Β *actin*	NM_031144.3	GCTACGAGCTGCCTGACGG	GAGGCCAGGATGGAGCC

Extraction and purification of the RNA were performed using the RNeasy Mini kit, cat. no.: 74,004 (Qiagen, Hilden, Germany). The procedure was carried out following the protocol described in the RNeasy Mini Handbook without modification. Up to 1 × 10^7^ cells were disrupted and homogenized by bead-milling in a guanidine-thiocyanate that includes lysis buffer, which instantly inactivates the RNAses to assure intact RNA purification. Then, ethanol was inserted to create a proper binding environment. The samples are then loaded onto the RNeasy Mini spin column. Total RNA was bound to the RNeasy silica membrane, and contaminants were eliminated efficiently by rinsing. The high-quality RNA was eluted in RNase-free water.

The reverse transcription (RT) phase was utilized by the QuantiTect reverse transcription kit, cat. No: 205,310 (Qiagen, Hilden, Germany). On the ice, 20 µL of reverse-transcription master mix was prepared. It was made up of 1 µL of QuantiTect reverse transcriptase enzyme, 4 µL of RT buffer, 1 µL of RT primer mix, and 14 µL of genomic RNA, so it has all the ingredients that are necessary for first-strand cDNA synthesis, except template RNA. The reaction mix was incubated for 15 min at 42 °C, then it was incubated for 3 min at 95ºC to inactivate Quantiscript reverse transcriptase. The RT reactions were placed on ice, and then real-time PCR proceeded directly.

The four genes O*cn*, R*unx2*, D*spp*, and C*ol1* were amplified from cDNA using the QuantiTect Syber Green PCR kit, Cat no: 204,141 (Qiagen, Hilden, Germany), and oligo-specific primer sequences. The thermal cycling conditions were as follows: 10 min at 95ºC, followed by 45 cycles of 95ºC for 10 s, 60ºC for 15 s, and 72ºC for 15 s. The condition of the melting curve analysis was 72–95ºC, increased by 1ºC per second. The 5-plex Rotor-Gene Q collected data automatically and analyzed the value of the threshold cycle (Ct), which normalized to an average Ct value of the housekeeping genes (∆Ct), and the relative expression of each representative was calculated as 2^–∆Ct^ [31].

### Statistical analysis

Numerical data were explored for normality and homogeneity by checking the distribution of data using the Shapiro–Wilk test of normality and Levene’s test for equality of variances. Data were presented as mean ± standard deviation (SD). ANOVA was used to test the total significance between more than 2 groups, followed by Tukey’s honest significant difference post-hoc test for pairwise comparisons. The significance level was set at *P* ≤.05. Statistical analysis was performed with IBM SPSS Statistics for Windows, version 23.0.

## Results

### Identification of DPCs

**Fig. 2:**
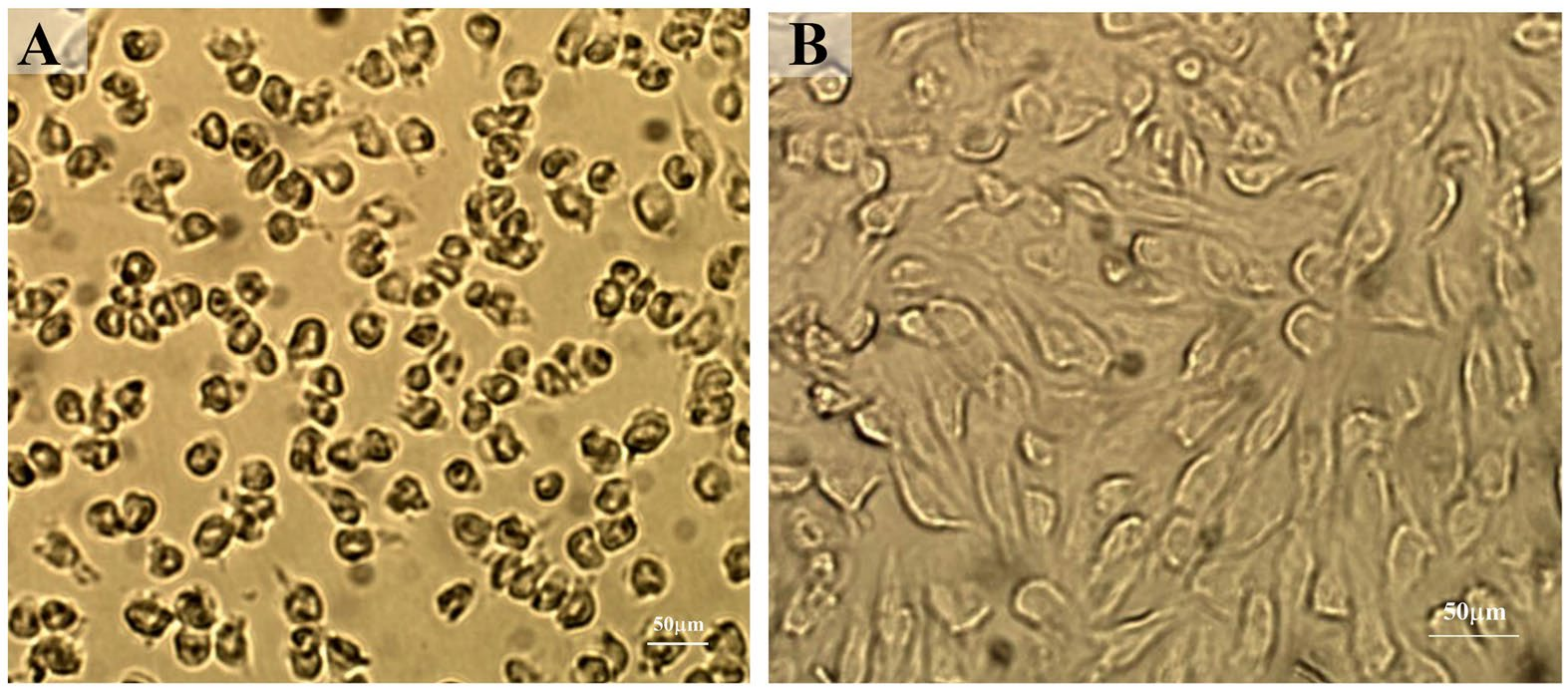
Inverted light microscopic representative images at 10× magnification showing rat’s DPCs on the 4th day (**A**); the great number of cells were rounded and nonadherent whereas on the 14th day (**B**); all adherent cells were spindle-shaped cells, Scale bar = 50 μm

Under the inverted microscope, the cells isolated from the rat’s dental pulp tissue were evaluated day after day till subculturing. At first, and on the 4th day, a great number of cells were rounded and nonadherent, whereas on the 7th day, there was an increase in adherent cells, and some rounded cells transformed into a fibroblastic spindle-like shape. Cell colonies were observed under the microscope on the 10th day, and on the 14th day, the confluence of cells was notified. At this point, all adherent cells transformed into spindle-shaped cells (Fig. 2).

### Characterization of DPCs

**Fig. 3:**
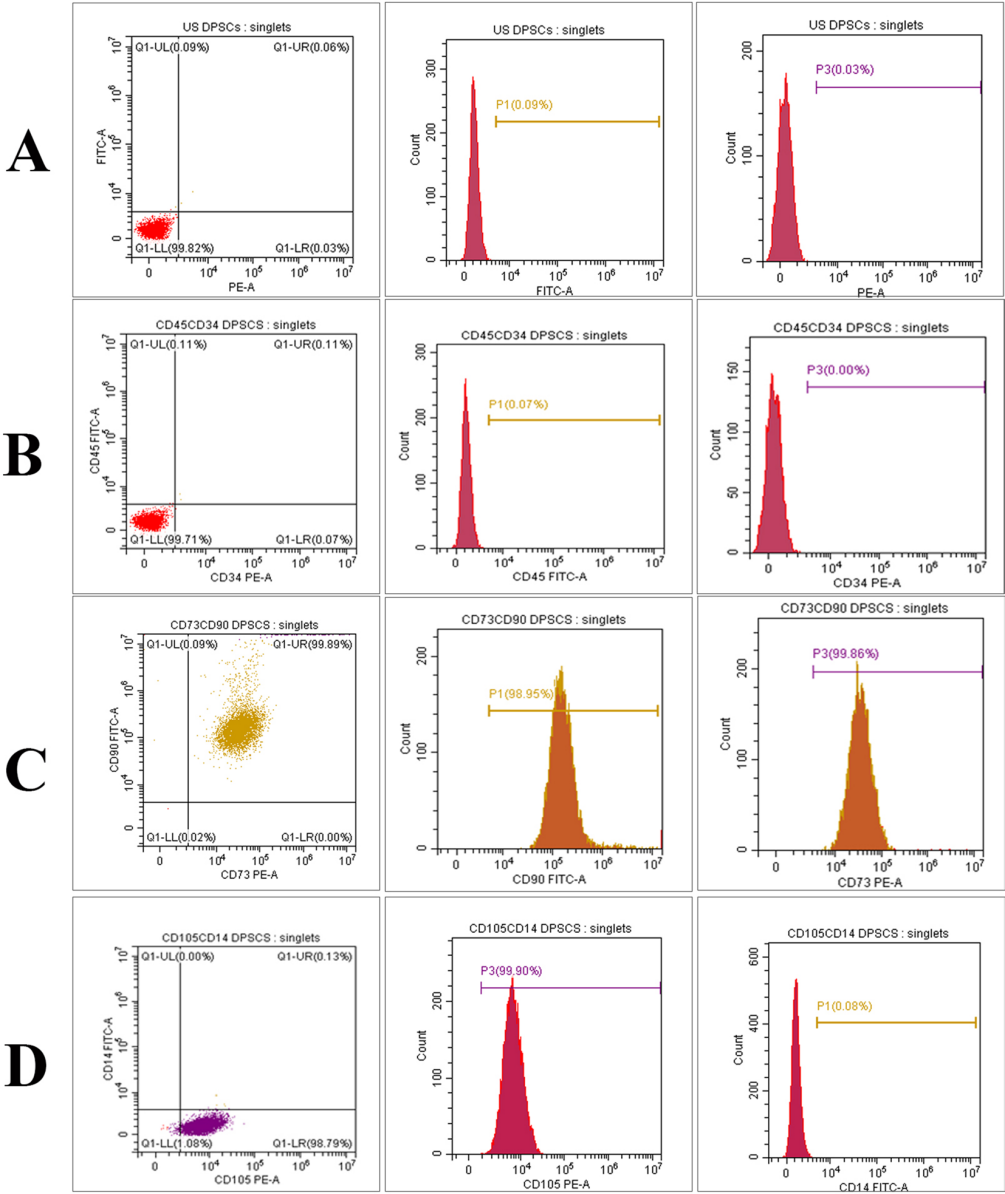
FC chart showing single parameter histogram for unstained DPCs (**A**), CD45 and CD 34 (**B**), CD90 and CD 73 (**C**), CD105 and CD 14 (**D**). FC analysis of rat’s DPCs showing the negative reaction of CD45 (0.07%), CD 34 (0.00%) and CD14 (0.08%) whereas the clusters of DPCs are positive to CD90 (98.95%), CD 73 (99.86%) and CD105 (99.90%)

The findings of the FC showed that DPCs were negative for CD45 (0.07%), CD34 (0.00%), and CD14 (0.08%), whereas the clusters of DPCs were positive to CD90 (98.95%), CD73 (99.86%), and CD105 (99.90%). These results reflect the high yield of mesenchymal stem cells isolated from rats’ dental pulp (Fig. 3).

### Nanoparticle tracking analyses and measuring sEVs concentration

**Fig. 4:**
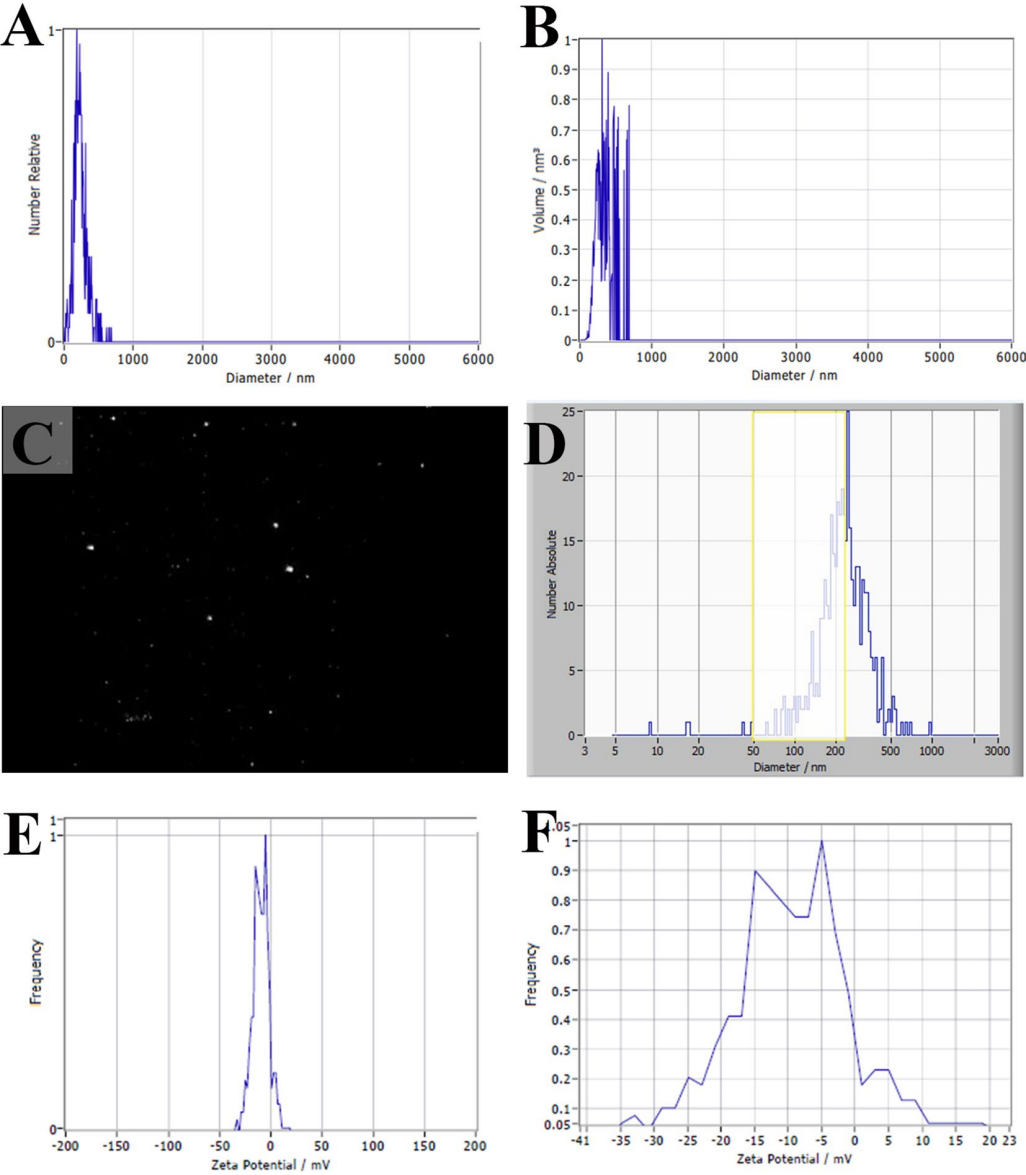
The particle size distribution was measured by NTA (**A**,** B**), cropped image from NTA video recorded by ZetaVIEW S/N 21–619 software (**C**), NTA particle distribution (Yellow box showed particle with size ranging from 50 to 225 nm) (**D**) and graphs showing Zeta potential measurement (**E**,** F**)

The NTA profiles of sEVs revealed that the major peak of sEVs was at 225.5 nm, i.e., 47% had an average diameter of less than 225 nm. Moreover, nanoparticle quantification confirmed that sEVs concentration was 6.2 × 10^7^ particles/mL. The surface charge of the vehicles revealed that 61.3% of sEVs zeta potential was − 6.5 mV while 38.7% exhibited − 11.5 mV, which indicated the stability of sEVs. Using the BCA protein assay, the total sEVs yield was determined by protein estimation from intact sEVs. Protein concentration from BCA assay: 4.2 mg/mL. The particle concentration from NTA analysis was 6.2 × 10^7^ p/mL, the dilution factor was 1, and the total volume of the medium was 400 mL. Therefore, the sEVs purity was calculated as 6.2 × 10^7^ (p/mL) x 1 × 400 (mL) / 4.2 (mg/mL) x 400 (mL), and the result was 1.4588235 × 10^7^ particles/mg protein (Fig. 4).

### Characterization of DPCs-sEVs by TEM. FC and WB

**Fig. 5:**
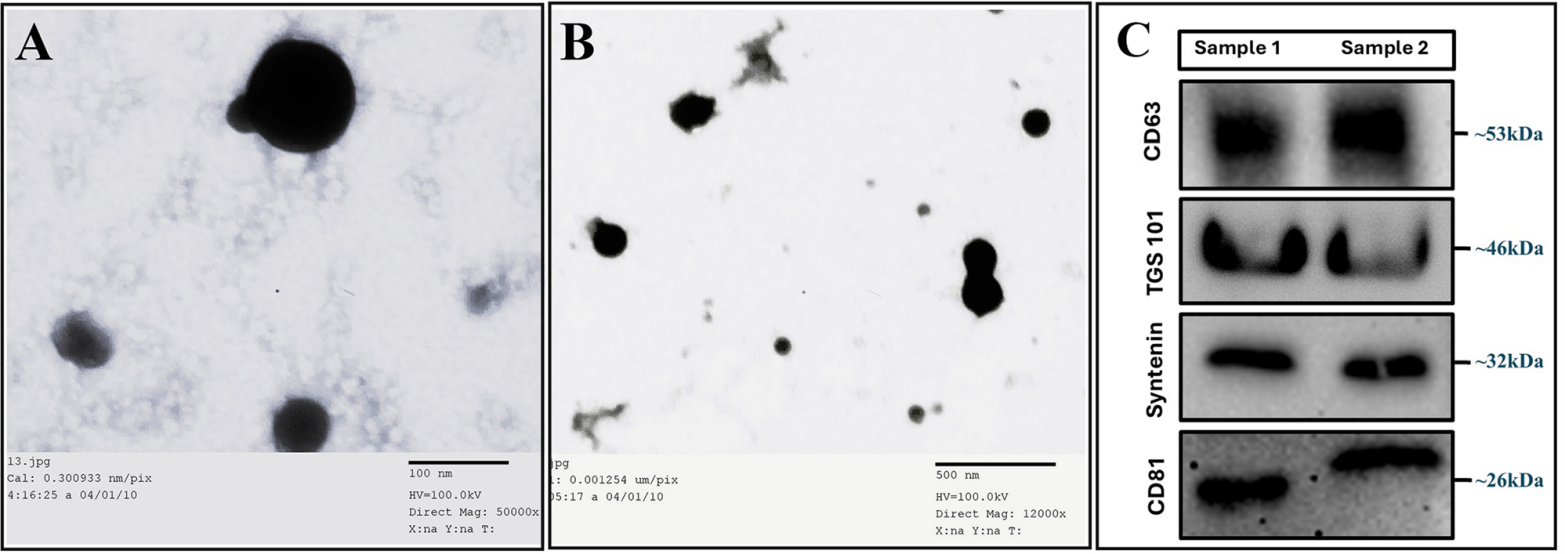
Compound image showing TEM images for the characterization of sEVs isolated from cultured rat’ DPCs. sEVs are wrinkled, oval or spherical under TEM, (**A**) 100 nm and (**B**) 500 nm scale bar, and (**C**) western blot images for duplicate sEVs samples showing CD63 tetraspanin protein with ~ 53 kDa, CD81 protein with ~ 26 kDa, TSG 101 Protien with ~ 46 kDa, and syntenin cytosolic protein with ~ 32 kDa

Transmission electron microscopy was used to confirm the presence of sEVs in the suspensions and can be used to describe sEVs’ morphology and size. sEVs shapes were wrinkled, oval, or spherical. The size of sEVs was 152.17 ± 47.5 nm in a range between 60 and 260 nm in diameter with a mean size of 230 nm measured by Image J software (Fig. 5A and B).

Flow cytometry was used to assess sEVs surface markers. The sEVs were stained with exosomes specific membrane markers (CD9 and HSP70) and CD34 as a stem cells marker. The expression for CD34 was negative (5.8%), whereas for CD9 and HSP70 it was positive, and they were 68.27% and 54.5%, respectively, which reflects pure sEVs isolation. Moreover, the TSG 101 (~ 46 kDa), CD81 (~ 26 kDa), CD63 (~ 53 kDa), and the cytosolic protein syntenin (~ 32 kDa) were identified using WB (Fig. 5C).

### Results of MTT assay

**Fig. 6:**
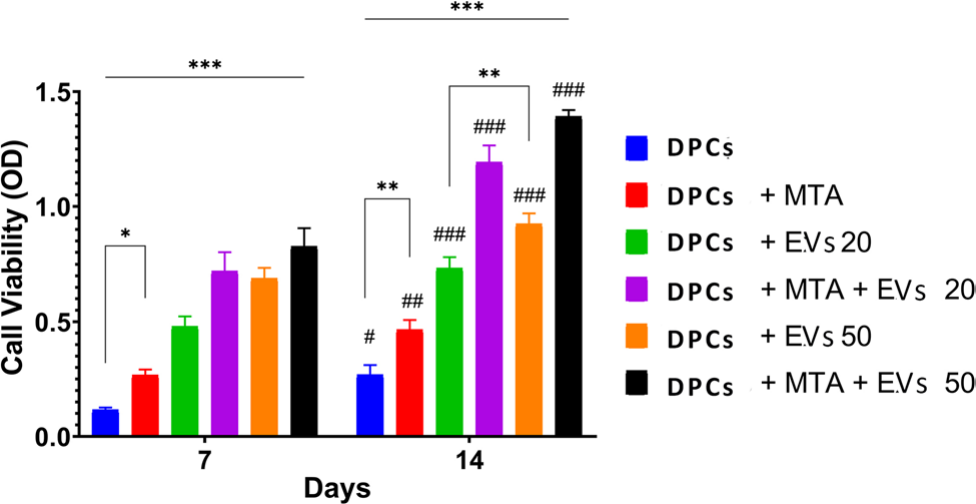
Bar graph representing mean values of MTT assay at 7 and 14 days’ time intervals. Results of MTT assay using two different concentrations of sEVs (20 and 50 µg/mL) with and without 0.2 mg/mL MTA combination and the DPCs alone mean values of MTT assay among tested groups. Statistical comparisons between groups treated for 14 days and 7 days are labeled with # symbols: # *p* <.05, ## *p* <.01, ### *p* <.001. Bars above groups marked with *** indicate significant differences between 7-day and 14-day treatments unless otherwise specified. Statistical significance within groups is denoted by * symbols: * *p* <.05, ** *p* <.01, *** *p* <.001

The MTT assay was used to detect the proper concentration of DPCs-sEVs. DPCs treated with 50 µg/mL DPCs-sEVs showed greater cell proliferation than those treated with 20 µg/mL either alone or in combination with MTA. Therefore, the optimal sEVs concentration selected in the present study was 50 µg/mL DPCs-sEVs (Fig. 6A).

Then, the MTT assay was used to determine the effect of sEVs, MTA, and their combination on DPCs proliferation rate after 7 and 14 days. The results showed that DPCs-sEVs, MTA, and their combination could stimulate the proliferation of DPCs, and the proliferative capacity of DPCs treated with the sEVs/MTA combination was significantly higher when compared with the other groups. After 7 days, the mean values of the MTT assay were 0.11 ± 0.00, 0.26 ± 0.02, 0.68 ± 0.04, and 0.82 ± 0.07 in the untreated group, MTA, 50 µg/mL sEVs, and MTA/50 µg/mL sEVs treated groups, respectively, and there was a statistical difference between all groups (*P* value < 0.001). After 14 days, the mean values of the MTT assay were 0.26 ± 0.03, 0.46 ± 0.03, 0.92 ± 0.04, and 1.39 ± 0.02 for the same groups, respectively, and there was a statistical difference between all groups (*P* value < 0.001). Moreover, the proliferation of DPCs after 14 days was greater than that after 7 days (*P* value < 0.001) (Fig. 6B).

### Results of transwell migration assay

**Fig. 7:**
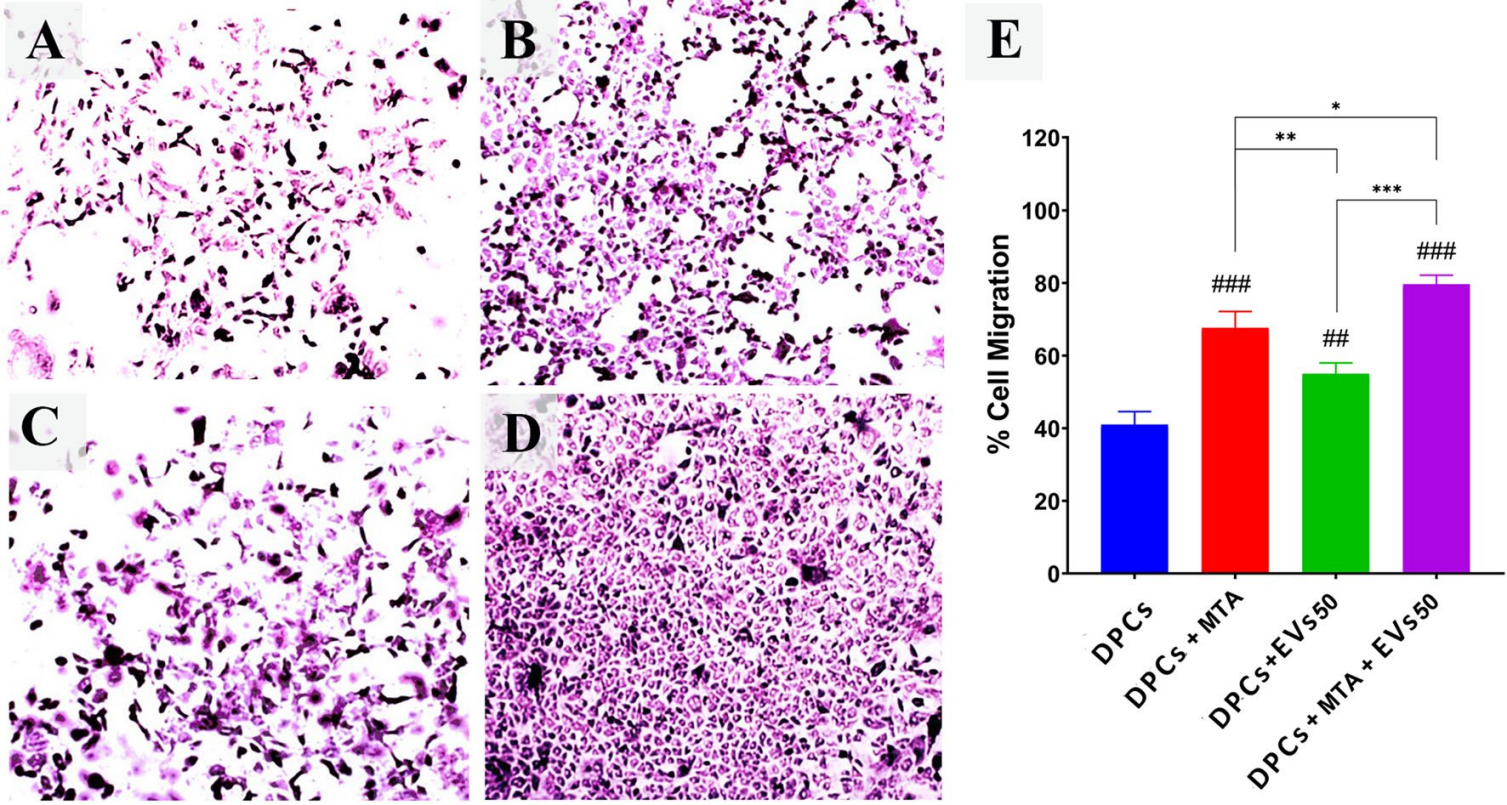
An in vitro transwell migration model was set up to analyze the ability of sEVs to induce site-directed migration of DPCs. **A**, **B**, **C** & **D** are inverted light microscope images showing the potentiality of control (**A**), 0.2 mg/mL MTA (**B**), 50 µg/mL sEVs (**C**) and the combination of 0.2 mg/mL MTA and 50 µg/mL sEVs (**D**) on DPCs migration (x10). (**E**) Bar graph representing % of migrated DPCs of transwell migration assay Statistical comparisons to the control group are labeled with # symbols: ## *p* <.01,### *p* <.001. Comparisons between treatment groups are indicated by * symbols: * *p* <.05, ** *p* <.01, *** *p* <.001

To determine the effect of MTA, sEVs, and their combination on the migration ability of DPCs, a transwell migration assay was performed. The results showed greater potentialities of DPCs migration in the treated groups compared to the untreated ones. MTA exhibited potentiality in inducing DPCs migration higher than untreated and sEVs treated groups. Moreover, DPCs treated with both MTA and DPCs-sEVs showed a significant increase in migration ability in comparison with other groups. The descriptive statistics for the mean and SD of the number of migrating cells revealed that the greatest mean value was for DPCs + MTA/50 µg/mL sEVs (79.00 ± 3.22), while the smallest value was for untreated DPCs (40.00 ± 1.79). The mean number of the migrated cells in the DPCs + MTA and DPCs + 50 µg/mL sEVs were 65.00 ± 2.68 and 53.67 ± 4.59, respectively. The one-way ANOVA test results showed that there was a significant difference (*P* =.001) among the various groups. Tukey’s honest significant difference test revealed a significant difference between the two groups (*P* =.001) (Fig. 7).

### Results of real-time polymerase chain reaction

**Fig. 8:**
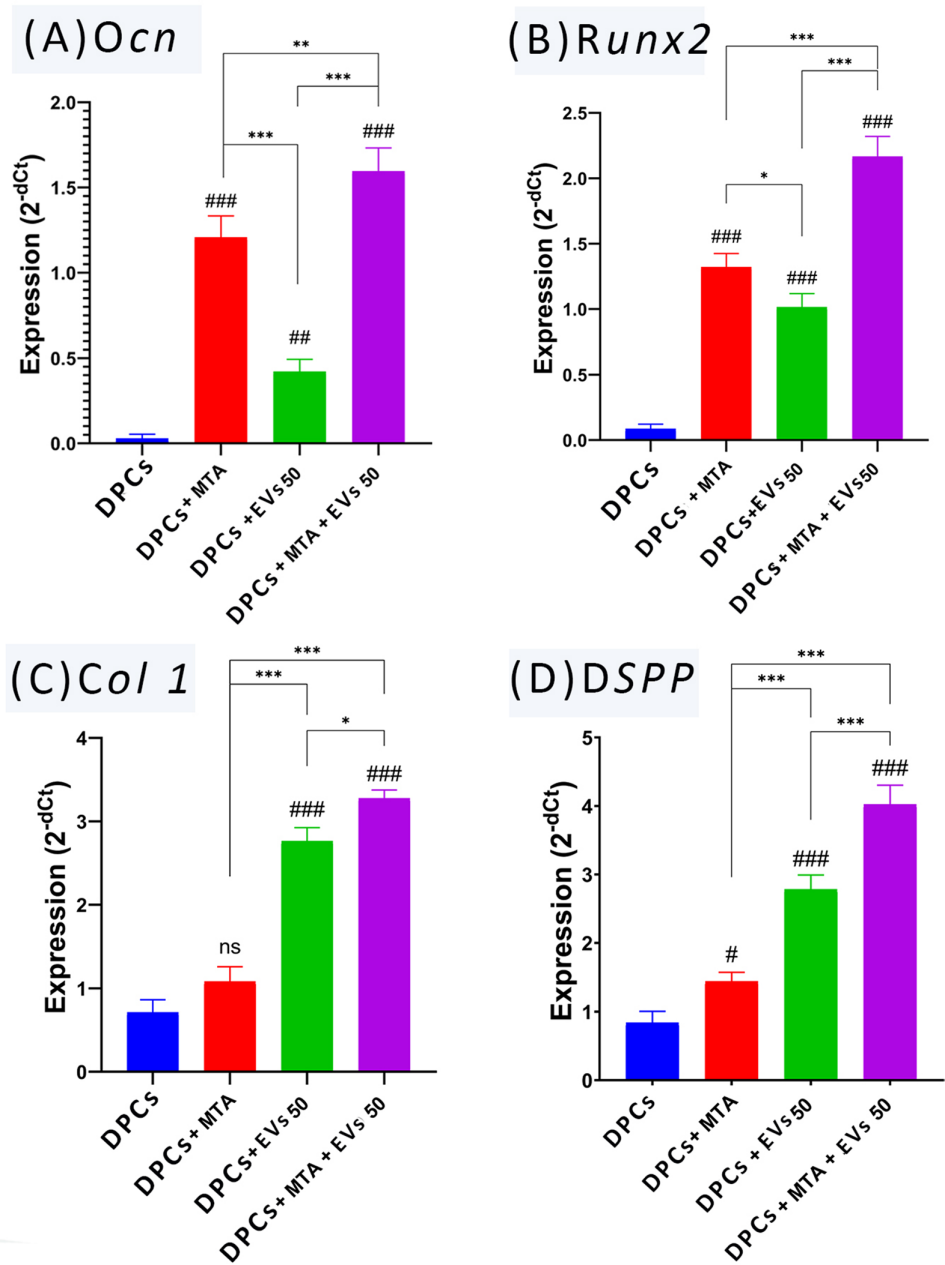
Bar graphs representing results of qRT-PCR for the amount of O*cn*, R*unx2*, *Col 1*, and D*spp*, and gene expression among tested groups, (**A**) statistical comparisons to the control group for O*cn* are labeled with # symbols: ## *p* <.01, ### *p* <.001. Comparisons between treatment groups are indicated by * symbols: ** *p* <.01, *** *p* <.001, (**B**) Statistical comparisons to the control group for R*unx2* are labeled with # symbols: ### *p* <.001. Comparisons between treatment groups are indicated by * symbols: * *p* <.05, *** *p* <.001, (**C**) Statistical comparisons to the control group for C*ol 1* are labeled with # symbols: **ns***p* >.05, ### *p* <.001. Comparisons between treatment groups are indicated by * symbols: * *p* <.05, *** *p* <.001, and (**D**) statistical comparisons to the control group for D*spp* are labeled with # symbols: # *p* <.05, *###p <.001.* Comparisons between treatment groups are indicated by * symbols: *** p <.001

Under the inverted microscope, the DPCs in the four groups were normal spindle-shaped cells without any abnormal observations. The RT-PCR was performed to measure the expression level of O*cn*, R*unx2*, D*spp*, and *Col 1* genes in the untreated and treated groups. Relative to B *actin*, the mean average of O*cn* for the untreated DPCs, DPCs + MTA, DPCs + 50 µg/mL sEVs, and DPCs + MTA/50 µg/mL sEVs were 0.03, 1.21, 0.421, 1.596, and for R*unx2* were 0.09, 1.32, 1.015, and 2.17 for the same groups, respectively. Whereas the expression patterns for D*spp* were 0.84, 1.44, 2.78, and 4.02, respectively. Moreover, the expression levels of C*ol 1*, were 0.71, 1.08, 2.76, and 3.28 for the same groups, respectively. One-way ANOVA test revealed a total significant difference between the mean averages of untreated DPCs, DPCs + MTA, DPCs + 50 µg/mL sEVs and DPCs + MTA/50 µg/mL sEVs (*P* =.001) for O*cn*, R*unx2*, D*spp*, and C*ol 1*. In addition, Tukey’s honest significant difference test revealed a significant difference between each two groups (*P* =.001). MTA treated group showed higher expression of the O*cn* gene than the DPCs-sEVs treated and untreated groups. MTA/50 µg/mL sEVs treated groups showed significant upregulation of both O*cn* and R*unx2* gene expression compared with other groups. The expression pattern of D*spp* and C*ol 1* was increased when using MTA or 50 µg/mL sEVs and 50 µg/mL sEVs showed higher expression in both genes compared with the MTA-treated group. The upregulated level was sharply elevated when utilizing MTA/50 µg/mL sEVs in comparison to other groups (Fig. 8).

## Discussion

Our aim was to evaluate the effect of MTA, sEVs, and the combination on DPCs’ viability, migration and osteo/odontogenic genes expression. The test groups showed different results with the superior results related to the combination group, therefore the null hypothesis of the present study was rejected.

Direct pulp capping involves capping the exposed vital pulp using a bioactive material to maintain the pulp’s viability and induce reparative dentin formation [32]. DPCs in dental pulp tissue are the main source of pulp healing/regeneration [33–35]. As the exposed pulp is covered by bioactive pulp capping materials, DPCs proliferate and migrate to the injured region to differentiate into odontoblast-like cells producing reparative dentin [34, 36, 37]. As pulp-dentin complex healing/regeneration needs DPCs, the adequate cells to be selected in our in vitro study were DPCs. Additionally, cell-derived sEVs have the same therapeutic benefits as their parent cells, so the selected cells used for sEVs isolation were also DPCs [38]. The DPCs-sEVs were isolated using the ultracentrifugation technique, which is the most common method for sEVs isolation. In addition, ultracentrifugation is suitable for separating most samples with low operating expenses [39, 40].

To evaluate the efficacy of sEVs, a commonly used pulp capping material is needed for comparison. The ideal pulp capping materials should have appropriate physical properties, sealing ability, antibacterial properties, biocompatibility, excellent handling, and a short setting time. Moreover, it must be able to regulate and promote the healing process brought on by DPCs [6]. To date, there are several materials used clinically for pulp capping, including Ca(OH)_2_ paste and MTA [5, 41]. MTA has generated considerable interest as a direct pulp-capping agent. Despite showing more effective and superior clinical and histological results compared to other pulp capping materials, its clinical application can be hampered by some drawbacks such as long setting time, high cost, slow degradation, and tooth discoloration [3, 9, 42]. For that, MTA was chosen to be used in our study as the commercial pulp capping material. As there are different types of MTA, we selected BioMTA (CERKAMED, Poland), which has complete biocompatibility, a lower setting time, three times higher hardness, and maximum plasticity [43].

An MTT assay was used to compare the effect of MTA, DPCs-sEVs, and MTA/DPCs-sEVs on cell viability and proliferation. Generally, cell viability assays are usually performed to assess the biocompatibility of cells after treatment. Our results showed that MTA and sEVs enhanced cell viability and proliferation, which confirmed their biocompatibility. This agreed with Babaki et al. [27] and Zhuang et al. [28], who found cell proliferation enhancement in the cells treated with 0.2 mg/mL MTA and 50 µg/mL sEVs, respectively [27, 28]. In line with Qiao et al., the use of two distinct sEV concentrations demonstrated that DPC-sEVs increased cell proliferation in a concentration-dependent way [44]. In contrast to the other groups in our investigation, the combination group exhibited the highest cell proliferation at the two distinct intervals, suggesting that the addition of sEVs improved the MTA biocompatibility and cell proliferation capacity.

The migrating behavior of DPCs is critical for pulp regeneration and repair. DPSCs migration and recruitment happen in the first phase of tissue repair during pulp capping [45]. Accordingly, it is critical to evaluate the effect of any potential pulp capping material on DPCs migration [46–48]. Our outcome showed that DPCs-sEVs enhanced DPCs migration. This result is supported by several research studies, such as Chen et al. [49] and Ivica et al. [50]. Ivica et al. [50] found that DPCs-sEVs enhanced the proliferation as well as migration of BMSCs, so it is suggested that sEVs can be used to recruit native DPCs to the desired site. Chew et al. [51] explained that sEVs enhance cell migration and proliferation via AKT and ERK signaling. Additionally, MTA-treated cells showed enhanced cell migration compared to untreated and sEVs treated cells. Our results are consistent with earlier research demonstrating that MTA might accelerate the migration and adhesion of DPCs, such as D’Antò et al. [52], Araújo et al. [53], and Seo et al. [54]. In our study, the MTA/DPCs-sEVs-treated cells showed superior enhancement in cell migration.

Several assays, including the alkaline phosphatase assay, alizarine red assay, and monitoring of marker expression using PCR, can be used to confirm osteo/odontogenic differentiation. One of the limitations of our study is that we solely used PCR to detect osteo/odontogenic differentiation. Several markers have been selected in the present study to be directly and indirectly involved in odontogenic differentiation. These include O*cn*, R*unx2*, D*spp*, and C*ol 1*. O*cn* was considered a marker of the late stages of osteo/odontogenic differentiation, which regulates the mineral phase of bone and dentin [55]. R*unx2* transcription factor is required for the activation of odontogenic signals, including B*mp4* and F*gf3* [56]. D*spp* is regarded as the specific marker for odontoblasts [57], and C*ol 1* is an odontogenesis-related protein [58]. In RT-PCR, MTA-treated cells showed higher expression of O*cn*, R*unx2*, D*spp*, and C*ol 1* than the untreated group. This result agrees with Zhao et al. [59], who investigated the effect of MTA on the odontoblastic differentiation from cultured DPSCs. It was shown that 0.2 mg/mL MTA significantly increased the expression of O*cn*, D*spp*, C*ol1*, alkaline phosphatase (A*lp*), and bone sialoprotein mRNAs. Wang et al. [60] evaluate the odonto/osteogenic capacity of inflammatory DPCs treated with MTA, and they found that MTA increased the expressions of O*cn*, R*unx2*, D*spp*, A*lp*, and osteoblast-specific transcription factor, which are odonto/osteogenic markers. They suggested that the NF-κB pathway plays a principal role during the differentiation of MTA-induced DPCs.

DPCs-sEVs-treated cells also showed upregulation of O*cn*, R*unx2*, D*spp*, and *Col1* genes expression than the untreated group. This result is supported by the findings of Huang et al. [61], who found that the expression of genes involved in odontogenic differentiation was elevated by DPCs-sEVs. Moreover, Zhuang et al. [28] reported that stem cells-derived sEVs induced BMSCs dentinogenesis in vitro as it upregulated the gene expression of D*spp*. They explained that BMSCs endocytose sEVs, and consequently, the dentinogenesis of BMSCs is enhanced, as mesenchymal stem cells derived sEVs include a variety of bioactive molecules that are transmitted to target cells and affect their tissue regeneration and fate. Moreover, the combination of MTA and sEVs showed superior results, indicating an enhanced effect.

Comprehensively, the results of the present study comply with the results of Shi et al. [62], who reported that dental pulp regeneration could be promoted by MSCs exosomes as it exerts a multi-faceted effect on DPCs functions, including odontogenic differentiation, proliferation, and migration. These cellular processes were enhanced through exosomal CD73-mediated adenosine receptor activation of AKT and ERK signalling. Using a rat pulp defect model, the authors found that MSCs exosomes promoted the formation of dentin-like tissue and bridge-like structures and increased the expression of dentin matrix proteins. In addition, following subcutaneous implantation in the mouse dorsum, MSCs exosomes yielded recellularized pulp-dentin tissues in the root canal of endodontically treated human premolars.

From the results of the present study and in the clinical situations of accidental pulp exposure, DPCs-sEVs could be used separately before using MTA pulp capping material. This enhances the regeneration capabilities of MTA as DPCs-sEVs promote healing towards regeneration. Together, they promoted cellular proliferation, migration, and O*cn*, R*unx2*, D*spp*, and C*ol1* gene expression. Our opinion is supported by Wen et al. [63], who evaluated the tissue responses and clinical outcomes of the dentin-pulp complex to the materials of treated dentin matrix (TDM), sEVs-TDM, sEVs, and MTA, and they found that the mineralized layers of dentin treated with MTA were thicker than those of the sEVs-TDM and the TDM-treated ones.

Conclusively, despite the current biological evidence displayed that the incorporation of bioactive components into the capping materials to target aspects likely as tissue inflammation and repair, more experiments are required to overcome shortcomings such as extensive significant cost, laboratory biological tests, and time implications. In addition, to carry these preclinical approaches to be effectively used in patient applications, academic collaboration in research with stakeholders such as global specialists are essential for targeting pulp capping material development.

## Conclusions

As demonstrated in this study, DPCs- sEVs were capable of enhancing cell proliferation, migration and odonto/osteogenic differentiation with superior results shown with the addition of MTA to DPCs-sEVs as a combination treatment. According to that, DPCs-sEVs can be considered as a potential pulp capping material, alone or in combination with MTA. As sEVs can be stored and commercially available, we can consider MTA/DPCs-sEVs combination as a potent combination for clinical advancement toward enhanced cell-free regenerative endodontics. Our results can be validated by further in vivo study using the same test groups to confirm the biological effect, biocompatibility, anti-inflammatory, and dentin bridge formation.

## Electronic supplementary material

Below is the link to the electronic supplementary material.

Supplementary Material 1

## Data Availability

The datasets used and/or analyzed during the current study are available from the corresponding author upon reasonable request.
