# Supplementary material for: Impact of dental pulp cells-derived small extracellular vesicles on the properties and behavior of dental pulp cells: an in-vitro study
Source: BMC Oral Health. 2025 May 10;25:704. doi: 10.1186/s12903-025-06031-0 (PMC12066064; doi:10.1186/s12903-025-06031-0)
Supplement: Supplementary file 1 — Supplementary Material 1 [file 12903_2025_6031_MOESM1_ESM.docx]

| Table S1: Antibodies used in western blot | | | | | | | | | |
| --- | --- | --- | --- | --- | --- | --- | --- | --- | --- |
| Antigen | **Vendor** | **Clone** | **Species** | **Cat. No** | **conditin** | **Dilution** | **MW [kDa]** | **Pos control** | **storage** |
| CD63 | biolegend | H5C6 | Mouse | 353013 | n.red. | 1:1000 | 53 |  | 4° C |
| CD81 | BD | JS-81 | Mouse | 555675 | n.red. | 1:1000 | 26 | HEK,THP-1 | 4° C |
| TSG101 | Sigma | 5701 poly | Rabbit |  | red./ n.red. | 1:1000 | 46 | HEK,THP-1 | ali-quotiert-20° C |
| Syntenin | abcam | EPR8102 | Rabbit |  | red. | 1:1000 | 32 | HEK,THP-1 | -20° C |

| **Table S2.** Components and ingredients of BIO MTA. | |
| --- | --- |
| **Components** | **Ingredients** |
| **BIO MTA powder** | Calcium oxide; hydroxyapatite; oxides of silicon, iron, aluminum, sodium, potassium, bismuth, magnesium, zirconium; calcium phosphate. |
| **BIO MTA liquid** | Purified water, calcium catalyst. |

**
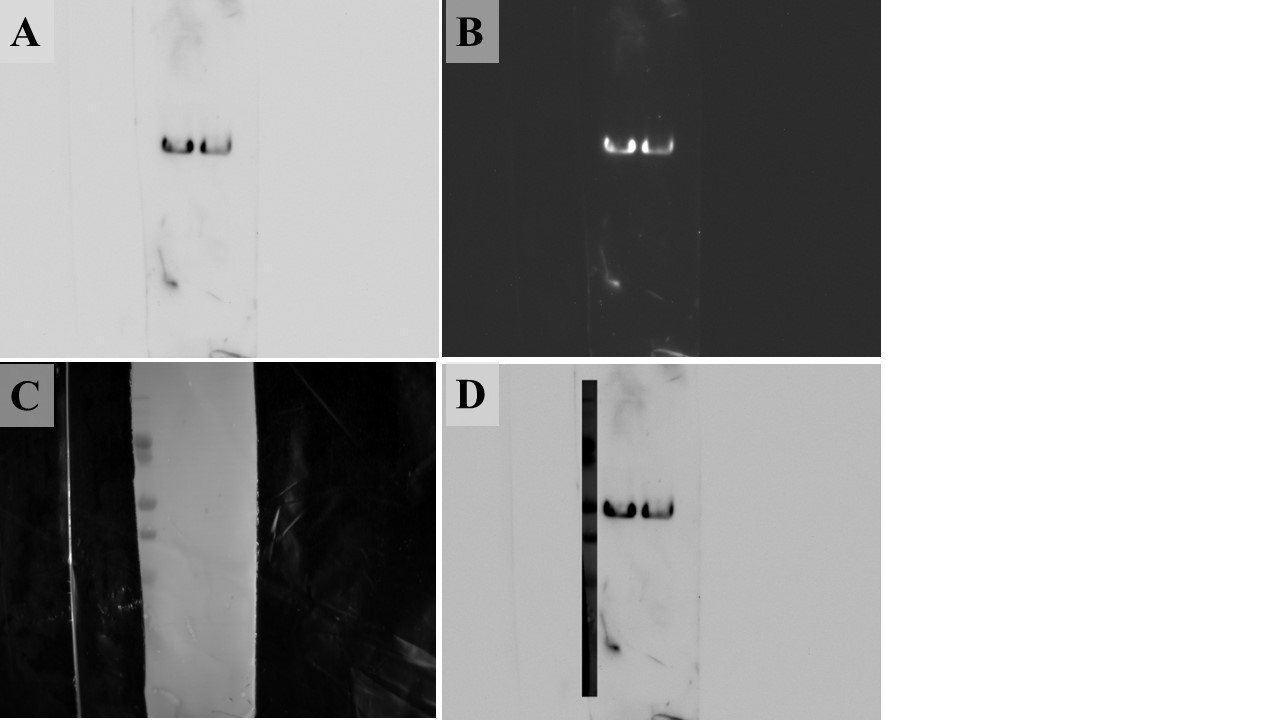
**

**Figure S1**: Uncropped western blot images for 2 sEVs samples showing TSG 101 Protein with ~46 kDa (**A**) non-inverted image, (**B**) inverted image (**C**) image for the ladder in the same membrane, and (**D**) merged image for the visualized bands in the membrane with the ladder. It was performed in Bernd Giebel Research Lab, Institute of Transfusion Medicine, University Hospital Essen, Germany.
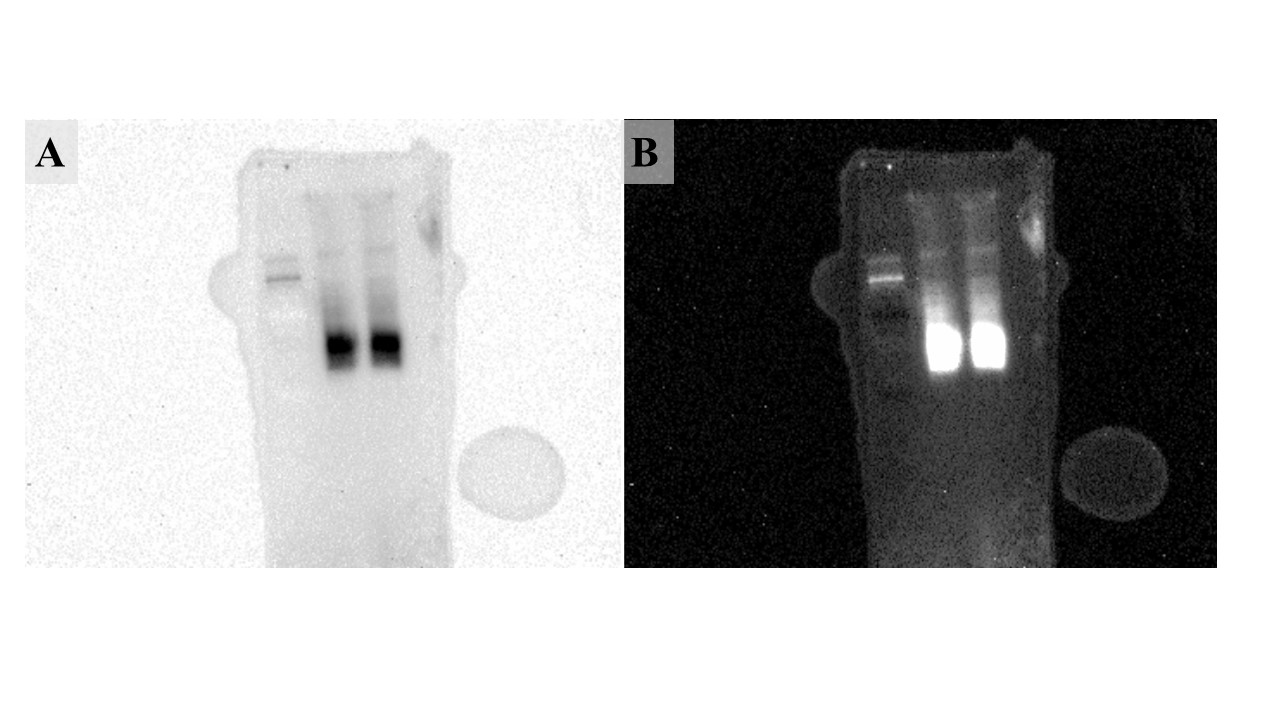
**Figure S2**: Uncropped western blot (**A**) non-inverted, and (**B**) inverted images for 2 sEVs samples showing CD63 tetraspanin protein with ~53 kDa. It was performed in Proteomics
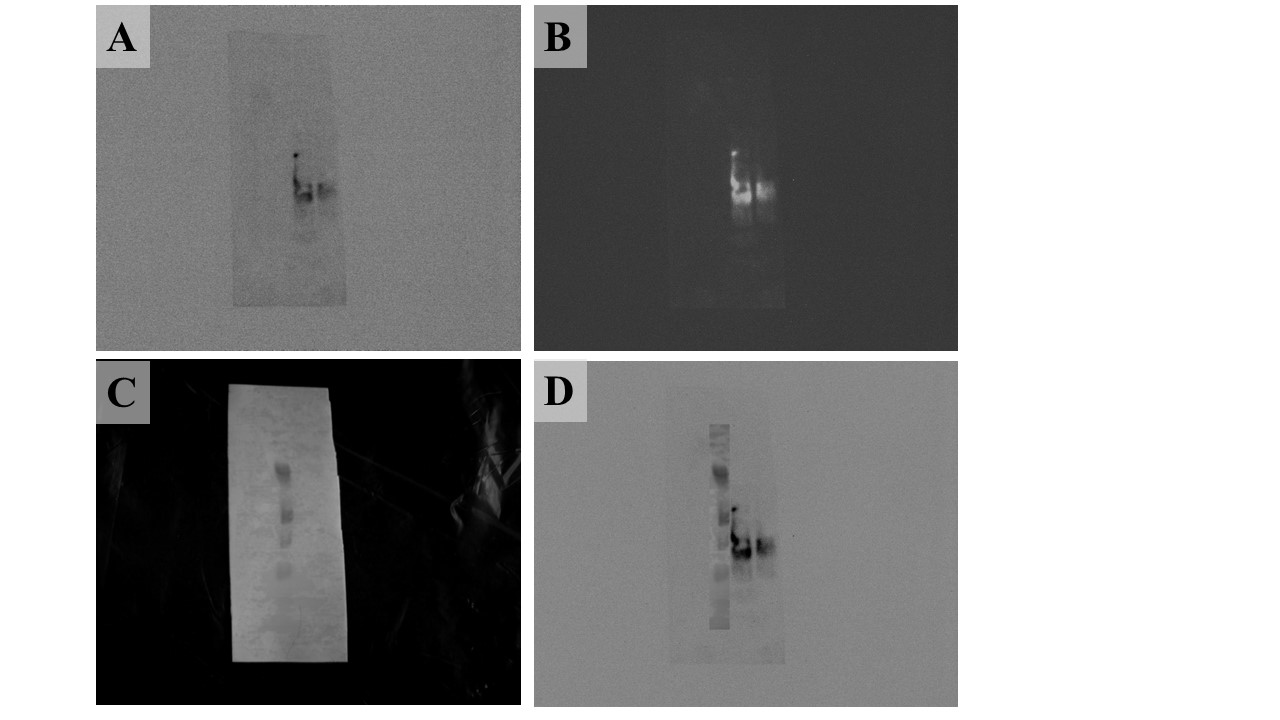
and Metabolomics unit, children’s cancer hospital 57357, Cairo, Egypt.

**Figure S3**: Uncropped western blot images for 2 sEVs samples showing CD63 tetraspanin protein with ~53 kDa (**A**) non-inverted image, (**B**) inverted image (**C**) image for the ladder in the same membrane, and (**D**) merged image for the ladder with the visualized bands in the membrane. It was performed in Bernd Giebel Research Lab, Institute of Transfusion Medicine, University Hospital Essen, Germany.
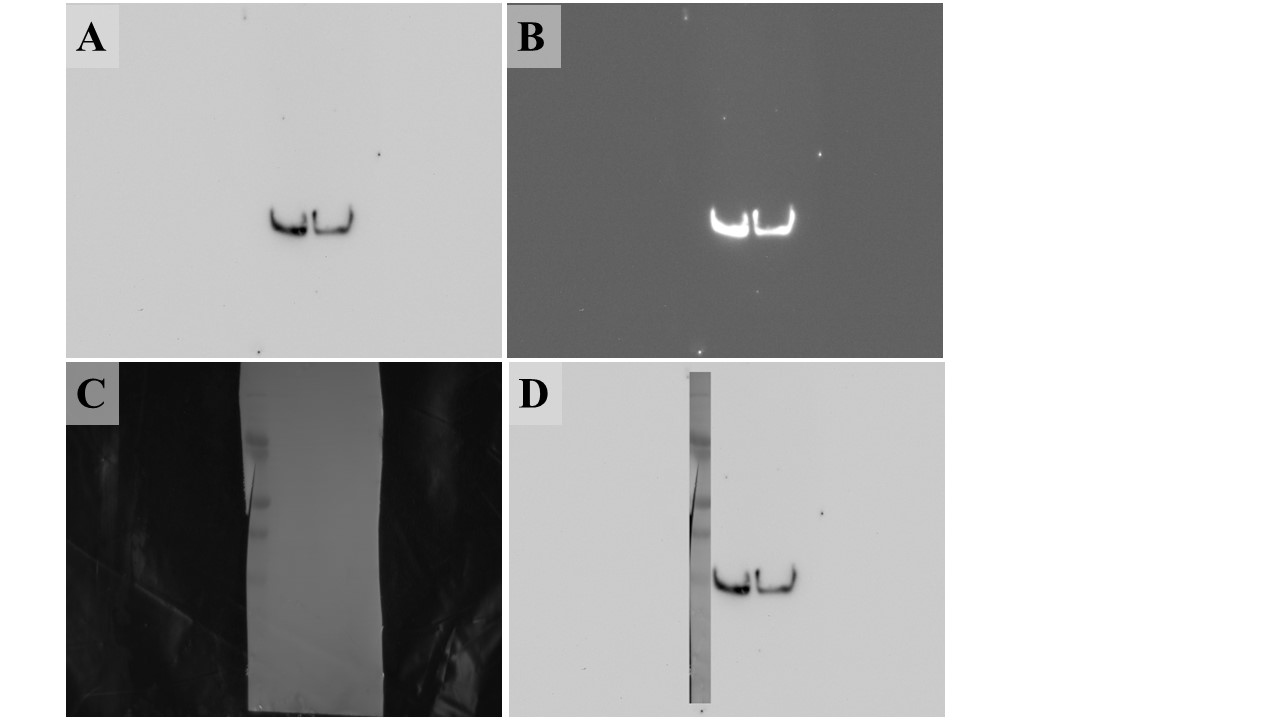

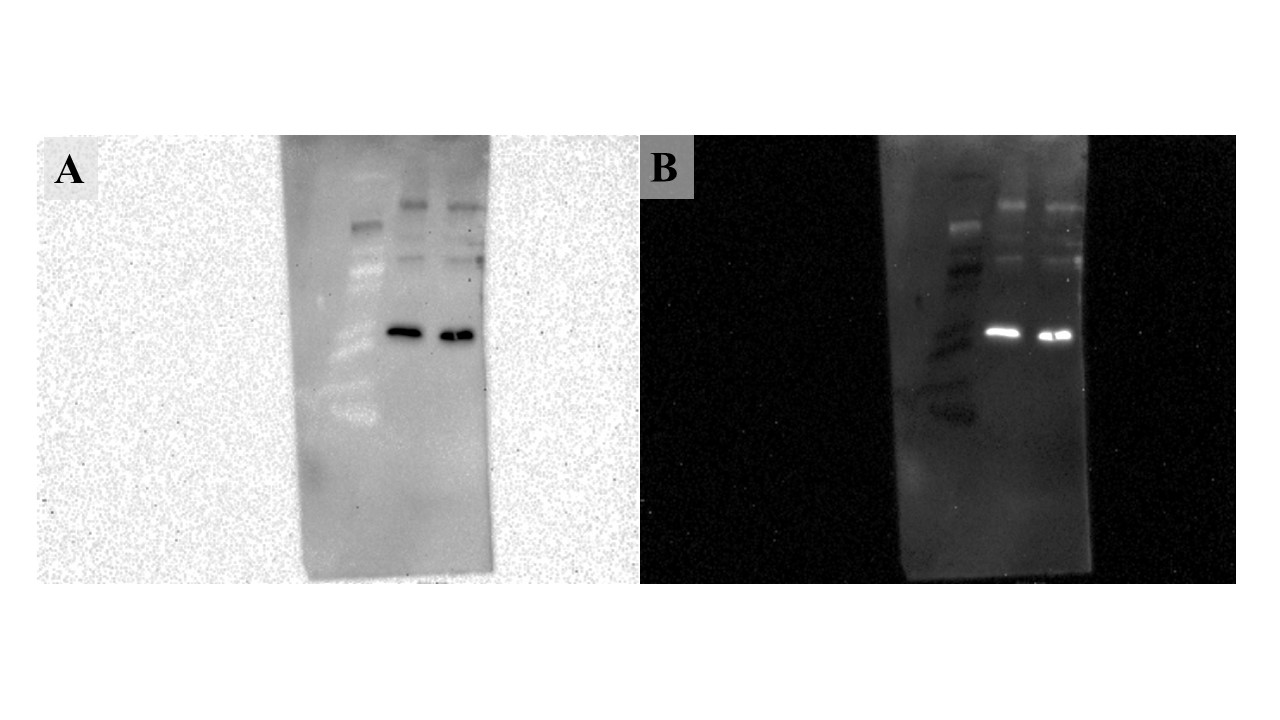
**Figure S4**: Uncropped western blot (**A**) non-inverted and (**B**) inverted images for 2 sEVs samples showing Syntenin cytosolic protein with ~32 kDa. It was performed in Proteomics and Metabolomics unit, children’s cancer hospital 57357, Cairo, Egypt.

**Figure S5**: Uncropped western blot images for 2 sEVs samples showing Syntenin cytosolic protein with ~32 kDa (**A**) non-inverted image, (**B**) inverted image (**C**) ladder, and (**D**) merged image for the ladder with the visualized bands. It was performed in Bernd Giebel Research Lab, Institute of Transfusion Medicine, University Hospital Essen, Germany.


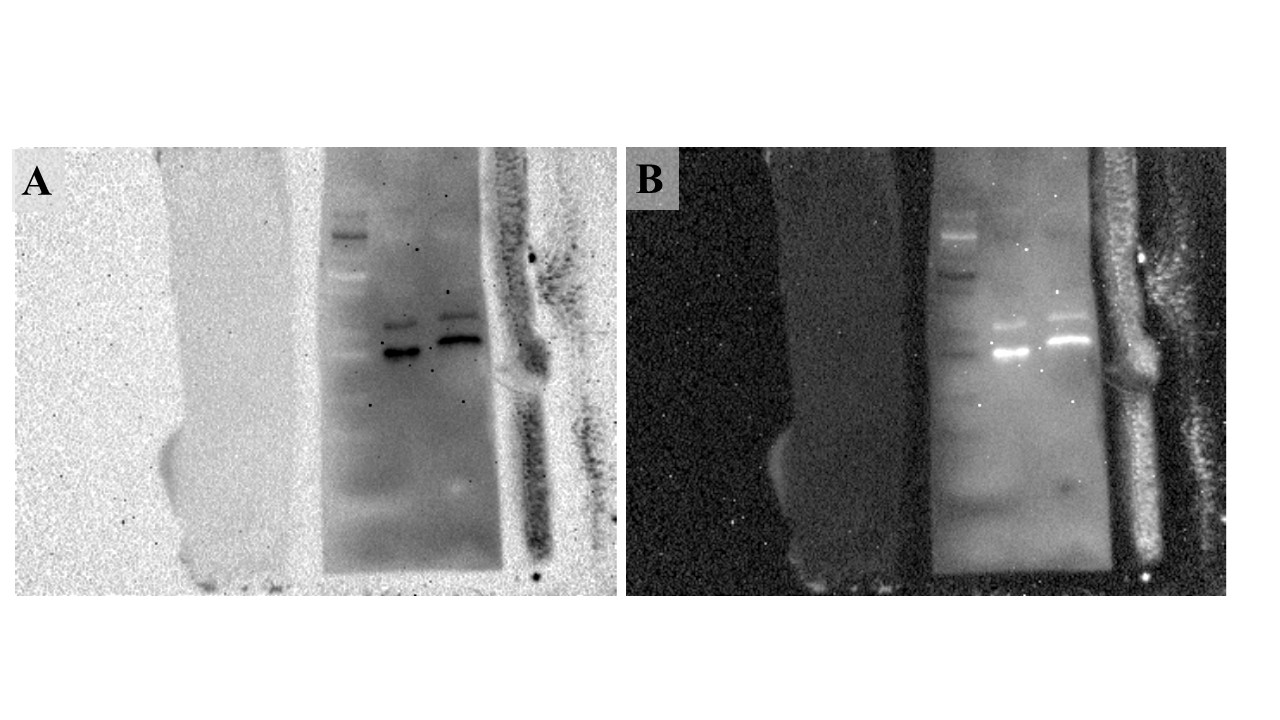
**Figure S6**: Uncropped western blot (**A**) non-inverted and (**B**) inverted images for 2 sEVs samples showing CD81 protein with ~26 kDa. It was performed in Proteomics and Metabolomics unit, children’s cancer hospital 57357, Cairo, Egypt.
